# Current research and evidence gaps on placental development in iron deficiency anemia

**DOI:** 10.1515/biol-2022-0827

**Published:** 2024-02-21

**Authors:** Shaoyang Lai, Weiwei Yu, Ying Liu, Yuxin Yang, Xueqin Zhang

**Affiliations:** Department of Obstetrics, School of Medicine, Women and Children’s Hospital, Xiamen University, Xiamen, China

**Keywords:** iron deficiency, maternal anemia, placental development, placental-associated disorders

## Abstract

Studying the effects of maternal iron deficiency anemia (IDA) is complex owing to its diverse causes, each independently impacting the placenta and fetus. Simple treatment with iron supplements does not always resolve the anemia. Therefore, delving into how IDA alters placental development at a molecular level is crucial to further optimize treatment. This review addresses the effects of IDA on placental structures and functions, including changes in oxygen levels, blood vessels, and the immune system. Profound understanding of physiological characteristics and regulatory mechanisms of placental development is key to explain the mechanisms of abnormal placental development in pregnancy-associated disorders. In turn, future strategies for the prevention and treatment of pregnancy complications involving the placenta can be devised. These studies are significant for improving human reproductive health, enhancing sociodemographic qualities, and even lifelong wellbeing, a focal point in future placental research.

## 1 Introduction

Recent evidence strongly shows that the intrauterine environment greatly influences the development of both the placenta and fetus. Placental structures and functions have been found to be altered in response to intrauterine conditions, including altitude hypoxia, maternal diabetes, and maternal anemia. These changes can lead to fetal growth retardation among other complications. They potentially represent placental adaptations, aimed at sustaining or optimizing its function amidst challenging conditions.

**Figure 1:**
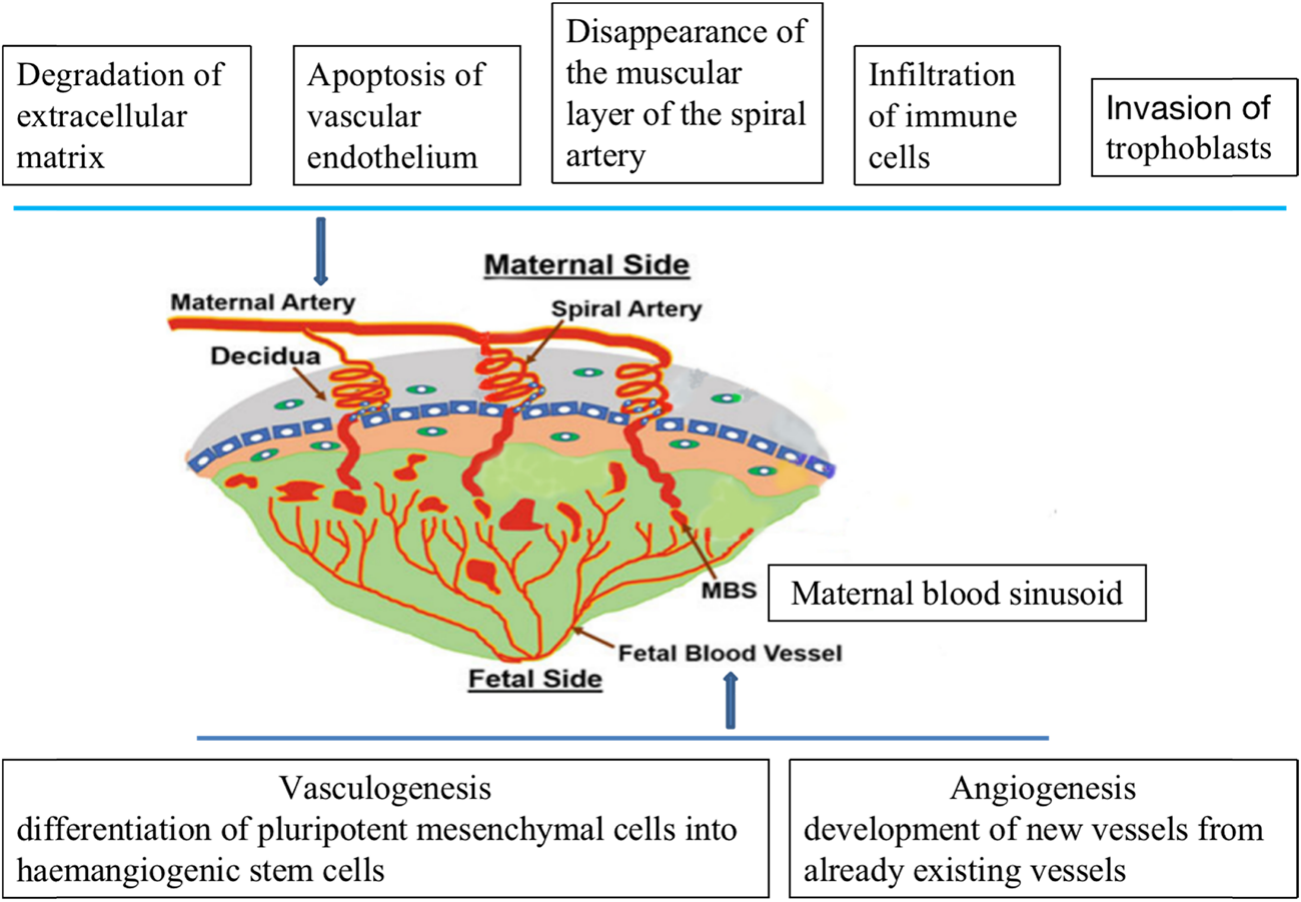
The latest theories of the placental development.

Iron is an essential nutrient for numerous biological processes. Physiologically, anemia is marked by the deficiency of hemoglobin content in red blood cells. Gestational anemia is mainly caused by maternal iron deficiency (ID) [1], attributed either to an unlikely cause of enigmatic deficiency of hemoglobin or mostly caused by a complex array of factors ranging from an orchestration of endogenous biological components including iron-elemental regulations to various exogenous supplementary, dietary, and socio-economic factors. Due to the high iron demand from the rapidly growing fetus and the expanding maternal blood volume, pregnant women are particularly vulnerable to ID. As per the World Health Organization, iron deficiency anemia (IDA) affects approximately half of the pregnant in developing countries [2], with Asia reporting the highest prevalence, with a percentage of 95% [3]. ID during pregnancy can result in serious risks for both the mother and her developing fetus. Iron-deficient mothers are at greater probability of requiring transfusion during parturition [4], and fetuses born to them have a higher risk of developmental delay, neonatal mortality, and morbidity [4]. Furthermore, infants born to these women could have long-lasting consequences with an increased risk of cardiovascular problems, diminished brain function, and compromised immune system development in adulthood [4]. The diverse causes of anemia complicate human studies into the effects of IDA, as different causes of anemia independently affect the placenta and fetus.

**Figure 2:**
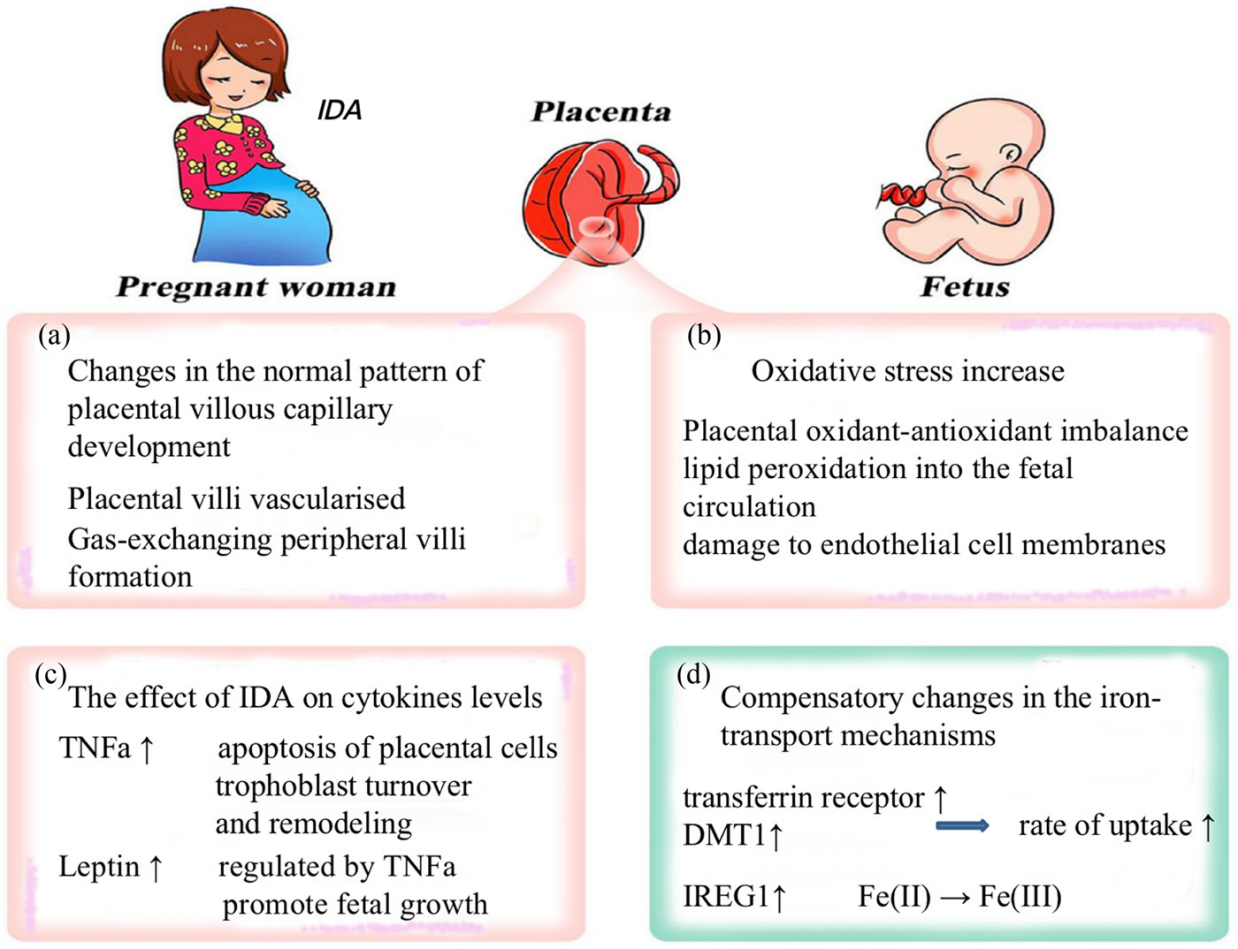
Proposed mechanisms by which IDA may influence the placental development.

The mechanisms underlying these effects have not been clarified, although it seems that early pregnancy development may play a pivotal role. During gestation, the growing fetus depends on its mother for nutrition and waste disposal across the placenta. The placenta is not merely a passive mediator; it can also regulate the rates and amounts of nutrient transportation as well as iron transportation. Consequently, IDA may affect the placental development and, subsequently, affect the development of the fetus. Several potential biological mechanisms have been identified through which anemia or ID could affect pregnancy outcomes. Anemia caused by hypoxia and ID can induce both maternal and fetal stress. Any stress-altering placental development or function is likely to have consequences for the developing fetus. Notably, high prevalence of anemia in pregnant women and present observations about limited treatment show that monotreatment with iron supplementation cannot clinically cure the anemia [5]. It is pertinent to peruse and discuss the various plausible reasons along with underlying factors contributing to such unfortunate but sizable burden. Thus, this article consolidates current research on the biological mechanisms involved in the development of the placenta and explores ways in which IDA impacts them. Next, it is important to explore how the placental development is altered by IDA, precisely the molecular basis behind this placental dysfunction, and then aim to optimize treatments for this problem.

## 2 The latest theories of the placental development

Since it is the communication interface between the mother and her fetus, the normal development of the placenta is crucial for the growth and sustenance of the fetus. The factors relevant to the course of placental development include gender, epigenetics, and external environment [6]. Gender affects the expression of sex hormone genes in male or female fetal placenta, respectively. For example, the STS gene, the sex hormone synthesis gene in X-linked genes, expresses in the female fetal placenta [7]; and the cluster of LHB-CGB gene also expresses those expression products such as luteinizing hormone and hCG related to the growth, invasion, and angiogenesis of placenta [8]. Epigenetics refers to the phenomenon of transmitting non-DNA sequence genetic information through mitosis and meiosis, including DNA methylation, histone modification, and non-coding RNA. It can play an important role in placental trophoblast cell differentiation, invasion, and hormone secretion by regulating gene expression. The reconstruction of methylation affects the early development of the placenta, for example, the inhibition of DNA methylation during early embryonic development disrupts the proliferation and migration of trophoblasts [9]. In addition, the growth of placenta requires a hyperoxic environment, because a decline in oxygen level will directly impair placental volume, development, and maturation by inducing increased activity of hypoxia-inducible factor (HIF) [10]. Understanding these changes during placental development and their effects on the varied physiological function are reviewed here, which is essential for elucidating the mechanism of embryonic development and the maternal and infant safety as well.

Placental formation is a very complex process, mainly comprising of spiral artery remodeling and placental angiogenesis [11]. The course of spiral artery remodeling involves extravillous trophoblasts retrogradely infiltrating maternal spiral arterioles under the guidance of various factors, gradually replacing the vascular endothelium, and then, the vascular muscle layer is replaced by fibrous substances. Finally, the lumen elasticity disappears with the decreased resistance and the blood volume increases [12]. Placental angiogenesis refers to increased formation of blood vessels at the fetal interface, which ensures that more placental lobules are in contact and material exchange occurs with spiral arteries [13]. Disruptions of either spiral artery remodeling or placental angiogenesis may lead to placental ischemia and placental oxidative stress, hence resulting in the development of various placental-associated disorders. Therefore, exploring each individual step during the development of the placenta and its regulatory mechanisms is beneficial to the further understanding of the pathogenesis and treatment of placental-associated disorders (Figure 1).

In the first trimester physiological hypoxia is essential for the development of placenta owing to the immature antioxidant capacity. Abnormal oxidative responses to peroxidation at early gestation, and hypoxia at late gestation or reoxygenation after maternal compensation, may impair trophoblastic invasion and placental capillaries formation, resulting in placental dysfunctions. The biological function of trophoblast changes because of the complicated regulatory mechanisms of hypoxia and hypoxia response in different stages of pregnancy. Therefore, further research on hypoxia and hypoxic response should not only clarify the regulatory mechanism of placental development but also reveal the pathogenesis of pregnancy-associated conditions and provides new clues for treatment.

Placental development has a lot in common with tumor progression [14]. The development of placenta, similar to malignant tissue behavior, starts with the proliferation of trophoblast cells in the uterus which profoundly affect maternal immune tolerance. Structural and functional changes in the placenta in pregnancy with IDA have been reviewed here, including issues regarding oxygen content, vascular components, and the immune system. Exploring placental development, physiologically and pathologically, enables researchers to have a deeper understanding of the relevant mechanisms of IDA and finally contributes to improving treatments and prevention of maternal and neonatal diseases.

## 3 Progress of clinical research on the placental development in IDA

The maintenance of pregnancy depends on the normal development and function of the placenta. Multiple factors that could influence placental development have been extensively investigated. Some scholars argue that placental weight tends to increase with increasing severity of the anemia, which has been widely interpreted as evidence of compensatory hypertrophy in response to a reduced oxygen supply [15]. Studies demonstrated that placental hypertrophy was associated with a mild and moderate degree of IDA, with no significant differences in volume fractions or surface densities of placental structures across anemic, intermediate, and non-anemic groups [15]. This enlargement of placenta was uniform with proportional growth of placental components. This conclusion was also supported by the significantly larger absolute volumes of intervillous space, non-intervillous space, or branch villi per placenta in the anemic group [15].

Conventionally, placental weight was regarded as an indicator of placental function. Stereological techniques have recently been applied to placentas from pregnancies exposed to long-term hypobaric hypoxia at high altitudes by measuring the values of key structural parameters to estimate the theoretic diffusing capacity of the organ [16]. Compared with lowland levels, the morphometric diffusing capacity is significantly increased at moderate altitudes despite considerable reduced growth of the fetal villous tree [16]. Although placental weight remained constant between the two groups there was a marked diminution rather than a hypertrophy in the villous tree in cases of maternal anemia [16].

Several large-scale studies have shown a notable negative correlation between maternal hemoglobin levels and placental sizes. IDA and maternal nutrition could influence the placental weight at birth [17]. Anemia itself rather than its type is the main factor of placental weight since there are no differences in their series comparing anemic women with thalassemia and those with IDA [18]. In some studies, there was not an association between hematological indices and placental size with discrepancies in study design and difficulty in standardizing the placental measurement at birth [19]. The maternal environment, more specifically maternal hemoglobin, could influence placental size in the mid-second trimester [20]. In some research, the placental volume was measured by three-dimensional (3D) ultrasound in the first and second trimesters [21], while similar placental measurement was obtained, they did not come to the same association between maternal hemoglobin levels and placental volume [22]. By 18 weeks of pregnancy, the placental volume may already be inversely correlated with maternal hemoglobin and serum ferritin concentrations, even in industrialized countries [22].

Contradictory studies have shown decreased villous surface area and a thinned harmonic mean thickness of the placental barrier [23]. Thus, they concluded that the placenta in the third trimester adapts to severe anemia by thinning the villous membrane so as to keep a normal diffusion capacity [23]. One study on term placentas from women with IDA has found a reduction in the size of the placental villous tree and a reduced thickness of the villous membrane. It meant that, overall, the diffusion capacity in these placentae was maintained despite the reduced size of the villous tree [23]. The results demonstrated that using placental size as an indicator of placental function can be unreliable since there may be changes in placental composition [23]. Another study has reported that placental vascularization increased in early gestation maternal anemia may reflect an adaptation to increased oxygen supply to the fetus [24].

In the light of the above observations, ID during pregnancy manifests a considerable variety of effects, culminating in placental dysplasia. Future fetal development depends on placental growth in early pregnancy and later differentiation might potentially have lifelong effects. Several reports suggest that inadequate prenatal growth patterns may be associated with problems later in life. At present, we cannot confirm that the outcome of pregnancy or later development will be compromised by ID, but future data will make it possible. Therefore, this review has good potential in advancing our understanding of how in utero nutrition relates to prenatal development.

## 4 Proposed mechanisms by which IDA may influence the placental development

Certain researchers propose that hypoxia might be the stimulus for the increased placental growth [25]. Some scholars have indicated that the levels of vascular endothelial growth factor (VEGF) stimulated by hypoxia correlate to the actual placental volume [13]. Moreover, it has been demonstrated that the maternal environment during the first and early second trimesters could affect placental growth.

However, further studies are needed to elucidate the physiological pathways involved.

The postulated biological mechanisms are outlined as follows (Figure 2).

### 4.1 Changes in the pattern of placental villous development in response to IDA

In the first trimester, placental villi undergo progressive vascularization by the process of branching angiogenesis within the stromal core under the influence of angiogenic growth factors, especially VEGF. Since VEGF transcription is upregulated in vitro by hypoxia, increased villous angiogenesis subsequent to maternal anemia may be mediated by an increased paracrine activity of VEGF [16]. Normal villous development during the second and early third trimesters is characterized by a gradual shift toward non-branching angiogenesis, resulting in the formation of gas exchanging peripheral villi [16]. Besides, a gradual decline in placental expression of VEGF and its FTR-1 receptor, in favor of increased placenta-like growth factor and KDR-receptor expression during pregnancy, may facilitate this switch to nonbranching angiogenesis [26]. Persistent branching angiogenesis could be the basis for the typical histological features of the anemic placenta at term [26] owing to “preplacental” hypoxia. Persistent branching angiogenesis within peripheral villi may therefore account for the epidemiological association between maternal anemia and placental characteristics [26].

### 4.2 IDA may increase oxidative stress

The feto-placental unit is highly susceptible to oxidative damage induced by reactive oxygen species. Placental oxidant-antioxidant imbalance might cause the release of products of lipid peroxidation into the fetal circulation with subsequent damage to endothelial cell membranes [27]. When the effect of ID on lipid peroxidation and antioxidant enzyme activity was examined in rats, the IDA group had significantly lower liver production of malondialdehyde and activity of superoxide dismutase and higher catalase activity [26]. The investigators speculated that the iron-sufficient rats were more able to eliminate oxygen radicals, whereas ID increased susceptibility to oxidative stress [26]. Moreover, erythrocytes are usually protected from oxidative stress caused by free radicals released from the potentially dangerous combination of iron and oxygen [26]. Since ID may increase oxidative stress, erythrocytes are more susceptible to oxidative damage [26]. Therefore, oxidative stress is one potential mechanism considered to cause pregnancy-induced hypertension, pregnancy-induced diabetes.

### 4.3 The effect of IDA on cytokine levels in placenta

Placental function is regulated, at least in part, by a broad spectrum of cytokines, both locally and distally. To our knowledge, placental cytokine levels are altered in ID. The changes are specific to different parts of the placenta, which may give some indication regarding the consequences of the alterations in levels [28]. The regulation of TNFα expression at the maternofetal interface must be crucial for successful placental development and function. TNFα is produced at low levels in placental and decidual immune cells during normal and healthy pregnancies, which is thought to be beneficial for pregnancy. It induces apoptosis of placental cells by the TNF-R1 pathway and, therefore, may be important in trophoblast turnover and remodeling [29]. Data also suggested that TNFα may regulate placental steroid production by the placenta and downregulate amino acid transfer [29]. Furthermore, the production of TNFα in IDA was increased by lipo-polysaccharide-stimulated mononuclear cells [29].

Leptin, which was first identified as a placental hormone in 1997 [30], still has unclear functions. Human studies have shown a correlation between cord blood and placental leptin but not between maternal leptin and birth weight [30], suggesting that placental leptin may promote fetal growth. Leptin secretion by adipocytes is regulated by TNFα, acting through TNF-R1 [30]. Whether this mechanism operates in the placenta has yet to be determined. Interactions between these cytokines are currently under investigation.

### 4.4 Compensatory changes in the iron-transport mechanisms of the placenta

ID in the maternal rat results in compensatory changes in the iron-transport mechanisms of the placenta, which, in turn, minimizes the level of ID in the fetus [31]. Most, though not all, of the proteins involved in iron transfer show increases at both mRNA and protein levels, commensurating with the increase in iron flux [31]. Transferrin receptor mRNA and protein levels increase both in iron-deficient placentas and in BeWo cells [32]. Increased transferrin receptor levels, concurrent with the rise in DMT1 will, however, imply that the rate of uptake into the syncytiotrophoblast will increase [32]. This would help compensate for the decrease in iron-transferrin concentrations in the maternal serum.

Similarly, the efflux mechanism also shows compensatory changes. At present, the best hypothesis explaining how iron enters into the fetal circulation is that it exits the cell through IREG1 ferroportin1 as divalent iron [33]. It is then oxidized to Fe(iii) by the copper oxidase and is incorporated into fetal transferrin. It would be expected that oxidase would be located close to IREG1 to facilitate the oxidation of Fe(ii) [33]. Besides, there are other proteins involved in the efflux pathway, probably both in the regulated expression of the actual transport proteins and also in the relation between the iron-regulatory proteins and the translation apparatus of the syncytiotrophoblast.

Interestingly, IREG1 mRNA levels do not change in iron-deficient placentas.

## 5 Discussion

Ever since the inception of the “Human Placenta Project” by the National Institutes of Health (NIH) in 2015, there has been a notable surge in placental research. Scholars can now visualize the live dynamics of the development of placenta, structurally and functionally, in physiological and pathological conditions by multidisciplinary collaboration. Their aim is to identify good biomarkers for predicting and diagnosing disorders in early pregnancy, while developing novel intervention strategies for pregnancy-associated complications by specifically targeting the placenta.

Some researchers believe that placental growth impairment and abnormal pregnancy adaptation might trigger excessive oxidative stress and placenta-derived cytokines acting on the circulatory system and exert permanent structural changes on the heart or arteries, which in turn may cause long-term cardiovascular diseases. Normal pregnancy is itself regarded as a physiological stress for mother. Abnormal pregnancy adaptation is not only one of the significant causes of maternal symptoms but also reflects certain maternal subclinical abnormalities, and clinical manifestations appear with pregnancy stress. The pregnant and parturient with pregnancy-related disorders are at high risk of developing other chronic diseases. Therefore, an appropriate and effective intervention and prevention strategy is needed.

Given the high prevalence of IDA, the profound subsequent complications, and the grim dilemma of treatment, greater emphasis should be paid to antenatal examination during pregnancy, early diagnosis, and timely treatment of IDA. This emphasis aims at promoting normal placental development, improving the current treatment effectiveness, and ultimately preventing adverse pregnancy outcomes. However, the available experimental evidence is still limited. There is an important bottleneck that our understanding of the regulation of human placenta development has not been discussed enough. Previous studies have proposed that placentas from IDA patients have physiological and pathological changes, including changes in the pattern of placental villous development, triggered oxidative stress, elevated levels of placental cytokines, and compensatory changes in the iron-transport mechanisms. Most studies at present are nearly cross-sectional, mainly focusing on the prevalence, the sociodemographic factors, the impact on maternal and fetal complications, and the efficacy of iron supplementation on IDA. The pathogenesis of IDA is mainly conducted on the change of iron conversion mechanism. The oral iron-supplement therapy is still the main clinical treatment of IDA, yet it might not consistently solve the problem. Consequently, the effective prediction and early diagnosis of diseases and then drawing up active and effective interventions is still unclear in this domain.

Prenatal anemia and ID are recognized as one of the preventable risk factors for disease with a substantial disease burden [34]. This calls for a rigorous evaluation of the effectiveness of existing antenatal care programmes in high-burden countries to identify gaps in policy and programme implementation. Targeted interventions to strengthen the infrastructure of antenatal care should be used. Future research to explore feasible strategies of ID in a country setting and evaluation of the effectiveness of other strategies, such as fortification and dietary diversification, should be explored.

Through the research on the mechanism of placental development, the current pregnancy management of IDA should be improved, and new treatment methods targeting the placenta should be devised to improve placental function and reduce the occurrence of long-term diseases of placental origin. However, there are still some limitations in the current research that are limited to animal models, lack of the support from large sample and multi-centre prospective research, and lack of a unified standard for the evaluation of placental function. And most studies are still in the basic experimental stage. Translation of these basic studies into clinical application will be the future research focus of placental medicine.

The effects of IDA on placental structures and functions by various factors have been reviewed, including changes in oxygen content, blood vessels, and immune system during the development of placenta. A deep insight into physiological characteristics and regulatory mechanisms of placental development was discussed, with the explanation of the mechanistic roles of abnormal placental development in pregnancy-associated disorders. Finally, strategies for the prevention and treatment of pregnancy-related complications targeting the placenta were explored. Investigating these aspects could vigorously promote great breakthroughs in the field of pregnancy maintenance and maternal–fetal health research. Such studies have crucial implications for improving human reproductive health, and sociodemographic factors, and even ensuring lifelong health maintenance. These remain pivotal areas of focus for our forthcoming placental research endeavors.
